# Association between Leukocyte Cell-Derived Chemotaxin 2 and Metabolic and Renal Diseases in a Geriatric Population: A Pilot Study

**DOI:** 10.3390/jcm12247544

**Published:** 2023-12-07

**Authors:** Aleksandra Kuzan, Krzysztof Maksymowicz, Emilia Królewicz, Karolina Lindner-Pawłowicz, Piotr Zatyka, Piotr Wojnicz, Maciej Nowaczyński, Adam Słomczyński, Małgorzata Sobieszczańska

**Affiliations:** 1 Department of Medical Biochemistry, Wroclaw Medical University, 50-368 Wroclaw, Poland; aleksandra.kuzan@umw.edu.pl; 2 Department of Forensic Medicine, Faculty of Medicine, Wroclaw Medical University, 50-345 Wroclaw, Poland; krzysztof.maksymowicz@umw.edu.pl; 3 Clinical Department of Geriatrics, Wroclaw Medical University, 50-368 Wroclaw, Poland; karolina.lindner-pawlowicz@umw.edu.pl (K.L.-P.); malgorzata.sobieszczanska@umw.edu.pl (M.S.); 4 Faculty of Medicine, Wroclaw Medical University, 50-367 Wroclaw, Poland; piotr.zatyka@student.umw.wroc.pl (P.Z.); maciej.nowaczynski@student.umw.wroc.pl (M.N.); adam.slomczynski@student.umw.wroc.pl (A.S.)

**Keywords:** LECT2, renal function, geriatric patients, metabolic diseases

## Abstract

LECT2 is not a routine diagnostic marker for any disease, but it has been associated with many pathologies, including systemic amyloidosis, rheumatoid arthritis, diabetes, atherosclerosis, and metabolic syndrome. With human aortic sections (*n* = 22) and sera from geriatric subjects (*n* = 79), we analyzed the relationships that could be observed between this protein and other parameters related to metabolic diseases. As a result, we observed a relatively high (r~0.8, *p* < 0.05) positive correlation between SRA and LECT2 and a negative correlation between EGFR and LECT2 (r~−0.4, *p* < 0.05). We observed LECT2 expression in macrophages, myocytes, and other aortic cells, with a tendency to be overexpressed in developed atherosclerotic plaques. We conclude that LECT2 exerts its chemotactic effects not only as a protein synthesized in the liver and secreted and circulating in the blood but also as a locally expressed protein within atherosclerotic plaque development. The LECT2-EGFR correlation suggests an association of this protein with loss of normal renal function. This fact can be associated with LECT2 amyloidosis, although it should be verified whether in the geriatric population there is indeed a widespread accumulation of LECT2 with the progression of aging or whether it is rather a marker of general deterioration of renal function.

## 1. Introduction

Leukocyte Cell-Derived Chemotaxin 2 (LECT2) is a hepatokine of the M23 family of peptidases [1]. It is a protein with pro-inflammatory functions; via its action as a chemotactic factor, it mainly affects the activation and migration of neutrophils toward the location of inflammation [2]. The chemotactic function of LECT2 is probably one of the factors involved in the pathogenesis of atherosclerotic plaque. This suspicion allows us to delve into the topic of metabolic syndrome as a cluster of cardiovascular risk factors that occur simultaneously in the same individual and is associated with systemic alterations that may involve several organs and tissues [3]. Biomarkers of the metabolic syndrome are potentially important diagnostic tools to maximize the effectiveness of treatment. It is worth emphasizing that the selection of biomarkers may be difficult due to the complexity of the syndrome. Neutrophils, which undergo chemotaxis toward inflammation, acting locally accelerate all stages of atherosclerosis formation via monocyte recruitment, macrophage activation, and cytotoxicity [4,5]. In addition, there was a positive correlation between high levels of neutrophils in the inner membrane of arteries and atherosclerotic plaque instability in humans and mice [4,6,7,8], which significantly increases the risk of a cardiovascular incident. Currently, one of the main risk factors for atherosclerosis is obesity [9,10]. In a study conducted by Okmura et al. [11], a positive correlation to elevated plasma LECT2 levels in obese patients regardless of gender, and a correlation of LECT2 levels to BMI and waist circumference were found.

A special group of patients who are dealing with atherosclerosis, as well as obesity, diabetes, and other problems generally referred to as metabolic syndrome are geriatric patients. We focused our attention on them, with the aim of preliminary analysis of the diagnostic utility of LECT2 in assessing the severity of metabolic diseases.

An additional goal of the project was to verify whether LECT2 is expressed inside the arterial walls where atherosclerotic plaque develops. Alternatively, it could be that LECT2 performs its action by interacting with the endothelium as a protein produced in the liver, from which it is released and circulates in the blood. The latter option is suggested, for example, by Protein Atlas, according to which this protein is expressed practically exclusively in the liver, in small amounts in the male testes, and in trace amounts in the retina [12].

Currently, many markers of kidney function are known. Among the biomarkers of kidney function, we can distinguish serum creatinine, urine output, and urine microscopy [13,14]. However, creatinine, as a marker for glomerular filtration rate (EGFR), is not sensitive, as it does not increase until a significant drop in EGFR or substantial parenchymal injury has occurred, nor does it represent any specific process that leads to the EGFR decline [15,16].

In our preliminary study, we tried to find a relationship between LECT2 and EGFR in order to develop a large-scale study in the future and develop a diagnostic model enabling the detection of kidney damage in the early (preclinical) phase, which could have important therapeutic consequences.

Moreover, we looked for correlations between LECT2 and non-routine biochemical parameters, such as advanced glycation end products (AGEs), galectin 3 (GAL3), and lipoprotein receptor-1 (LOX1) protein content, soluble receptors for advanced glycation products (sRAGE), scavenger receptor class B (SR-B), and scavenger receptor class A (SR-A) protein involved in the pathomechanism of metabolic syndromes.

## 2. Materials and Methods

### 2.1. Research Materials

One of the study materials was the sera of 79 patients hospitalized at the Department of Geriatrics, University Teaching Hospital, in Wrocław. Participants in the study signed written informed consent, and the study was approved by The Bioethical Commission at Wroclaw Medical University (opinion number KB-344/2017, approval date 26 May 2017). The inclusion criteria for the study group were age over 60 and the ability to express informed consent in the study. The exclusion criteria were acute conditions 4 weeks prior to study participation, such as myocardial infarction, stroke, venous thromboembolism, and cancer. Samples were collected between 7 June 2017 and 24 September 2019.

The results of routine laboratory tests were used, such as serum levels of glucose, glycated hemoglobin (HbA1c), cholesterol, HDL, LDL, triglycerides, creatinine, and uric acid, as well as estimated glomerular filtration rate (EGFR). In addition, the results of a project published in 2022 were also used for the analysis [10], that is, the content fluorescent forms of AGEs, GAL3 and LOX1 protein content, sRAGE, SR-B, and SR-A protein content. The methodology for the above-mentioned non-routine biochemical parameters was described in [17].

The second research material was fragments of the human thoracic or abdominal aorta. The study was approved by the Bioethics Committee of Wroclaw Medical University (opinion number KB-577/2017, approval date 26 May 2017). The samples came from 22 people who died in the sudden death range of 68 ± 15 years old. The inclusion criteria for the study group were death between 24 and 48 h before material collection and no signs of decomposition or mechanical destruction of tissues. The exclusion criteria were decomposition or mechanical destruction of tissues. The material was collected during forensic autopsies carried out at the Department of Forensic Medicine at the Wroclaw Medical University between 12 December 2017 and 30 June 2019.

### 2.2. Analysis of LECT2 Protein Content in Serum—Immunoenzymatic Assay

The concentration of LECT2 in serum samples was performed using human immunoassay ELISA kits from ELK Biotechnology (ELK Biotechnology Co., Ltd., Wuhan, China). The manufacturer’s instructions were followed.

### 2.3. Analysis of LECT2 Protein Content in Tissue—Immunohistochemistry

Paraffin slides with aorta sections were deparaffinized in xylene, rehydrated in decreasing concentrations of ethanol (100–50%), rinsed in PBST, and then antigen retrieved using HistoReveal (Abcam, Cambridge, UK). The preparations were treated with Bloxal Blocking Solution (Vector Laboratories, Inc., Newark, CA, USA) to block endogenous peroxidase and Normal Horse Serum (Vector) to block non-specific sites. Then, the preparations were incubated with the primary anti-LECT2 antibody (Abcam, Cambridge, UK) for 18 h at 4 °C. This was followed by a reaction with biotinylated secondary antibodies (ImmPRES Universal Polymer reagent, Vector Laboratories, Inc.) and with the ImmPACT DAB EqV Reagent substrate (Vector Laboratories, Inc.). Then, staining of basophilic structures with hematoxylin, rinsing with tap water, dehydration with increasing concentrations of ethanol (50–100%), preservation in xylene, and sealing with DPX (Aqua Medica, Lodz, Poland).

Primary anti-LECT2 antibody (Abcam, Cambridge, UK) was used.

### 2.4. Statistical Analysis

Statistical analysis was conducted using the data analysis software system Statistica (version 13.3, StatSoft, Hamburg, Germany). Relationships between parameters were analyzed using Spearman correlation. The effect of various factors (myocardial infarction or stroke, hyperlipidemia, hypertension, diabetes mellitus, hepatic steatosis, polyneuropathy, limb atherosclerosis, chronic heart failure, insulin treatment, and metformin treatment) on serum LECT2 content was analyzed using the Mann–Whitney *U* test (with correction for continuity). Statistical significance was taken at *p* < 0.05.

## 3. Results

The mean serum LECT2 content of the study group was 63.1 ng/mL, with a relatively high standard deviation of 50.2, indicating the high variability of LECT2 concentrations in the geriatric population. Analyzing the associations between this analyte and other characterized blood parameters, we observed a positive correlation between SR-A and LECT2 and a negative correlation between EGFR and LECT2. The association between LECT2 and creatinine was also close to statistical significance, which, together with the LECT2–EGFR correlation, confirms the relationship between renal function and LECT2 levels. Details of the magnitude of the correlation coefficient (r) and probability value (*p*) are presented in Table 1.

It turned out that there was no statistically significant relationship between the serum LECT2 content and diseases and conditions such as heart attack or stroke, hyperlipidemia, hypertension, diabetes, fatty liver, polyneuropathy, atherosclerosis of the limbs, chronic heart failure, insulin treatment, and metformin treatment.

**Figure 1:**
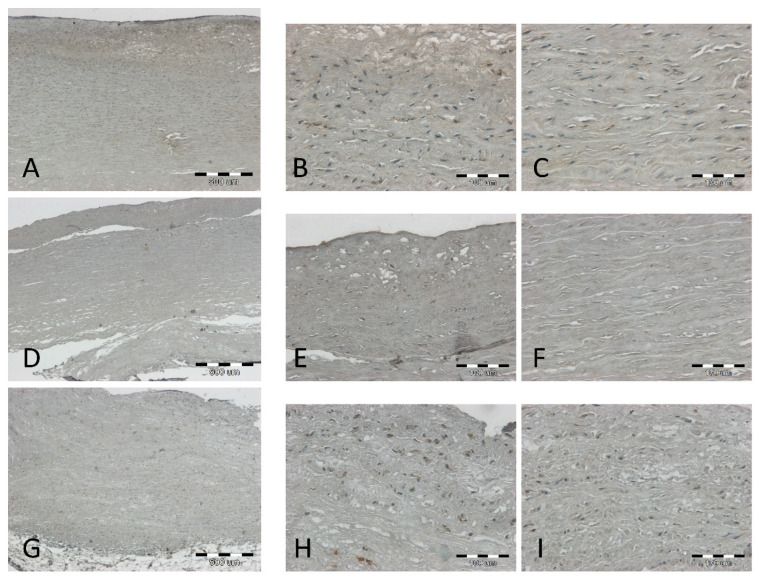
Results of immunohistochemical staining (IHC) for LECT2 of the aorta at low and intermediate stages of atherosclerosis—three representative cases (**A**–**C**; **D**–**F**; **G**–**I**) with 40× (**A**,**D**,**G**) and 200× magnification (**B**,**C**,**E**,**F**,**H**,**I**). The scale bar on panel (**A**,**D**,**G**) is 500 µm, and the scale bar on panel (**B**,**C**,**E**,**F**,**H**,**I**) is 100 µm.

**Figure 2:**
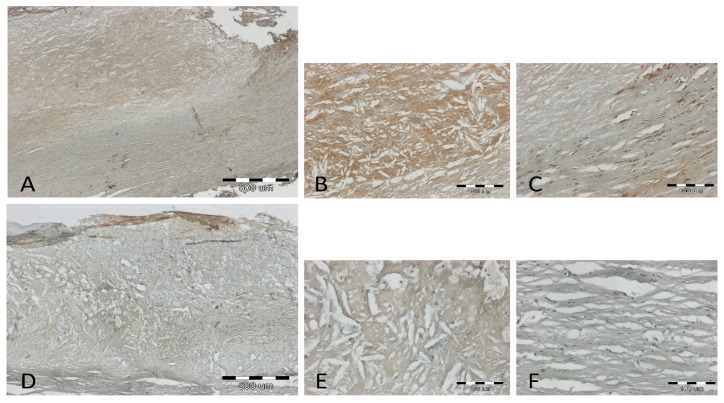
Results of immunohistochemical staining (IHC) for LECT2 of the aorta at a high stage of atherosclerosis—two representative cases (**A**–**C**; **D**–**F**) at 40× (**A**,**D**) and 200× magnification (**B**,**C**,**E**,**F**). The scale bar on panel (**A**,**D**) is 500 µm, and the scale bar on panel (**B**,**C**,**E**,**F**) is 100 µm.

**Figure 3:**
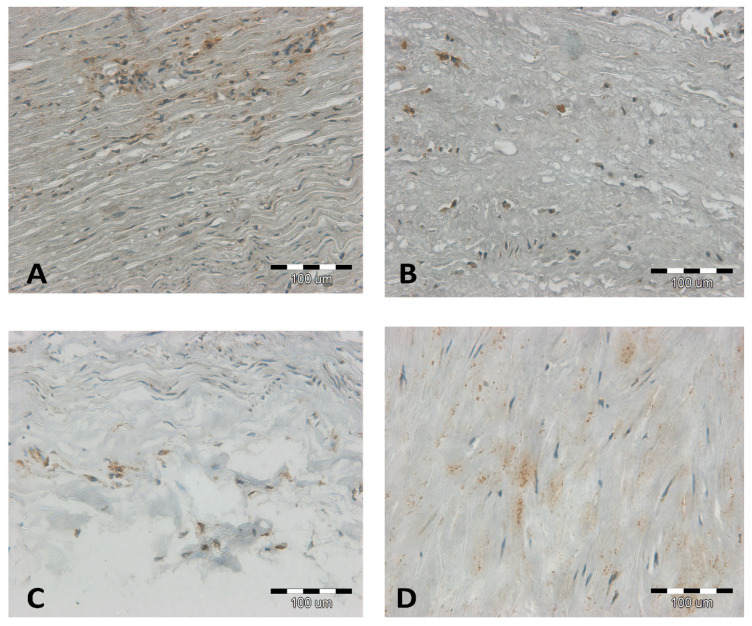
Results of immunohistochemical staining (IHC) for LECT2 of the aorta at 200× magnification (**A**–**D**)—representation of types of localization in cells. The scale bar on panel (**A**–**D**) is 100 µm.

It was observed that at low and intermediate stages of atherosclerosis (stage I–IV on the 6-degree scale of H. Stary), the LECT2 antigen is visible and colocalizes around the nuclei of various cell types: macrophages, myocytes, and adipocytes (Figure 1). In samples with very advanced atherosclerosis (stages V and VI on H. Stary’s 6-degree scale), the density of the antigen in the tissue is usually higher, especially in the atherosclerotic plaque, but also in the cells of the media (Figure 2). Thus, we confirm that LECT2 is expressed inside the aorta. Figure 3 collects the different types of localization of the antigen in the tissue: in Panel A, it is present in cytokine-activated migrating myocytes scattered in the cytoplasm; in Panel B, it is present in macrophages transforming into foam cells closely around the nucleus; Panel C shows the boundaries between the media and the tunica *adventitia*, with the antigen also observed in macrophages; Panel D shows a compact medium, with the antigen scattered throughout the cytoplasm of myocytes.

## 4. Discussion

As mentioned in the introduction, Protein Atlas reported that LECT2 is expressed almost exclusively in the liver [12], while our study showed that LECT2 is also present in the cytoplasm of macrophages and myocytes and is particularly concentrated in very advanced atherosclerotic plaques. Our result is consistent with the data obtained by Nagai et al., who also found LECT2 expression in vascular, endothelial, smooth muscle cells, adipocytes, and a few more cell types based on immunohistochemical analysis. Furthermore, the authors report that tissues that generally did not have LECT2 expressed at baseline included osteoblasts, chondrocytes, cardiac tissue, smooth muscle cells in the gastrointestinal tract, and the epithelial cells of some tissues—the LECT2 expression increased during disease conditions [18,19].

Nasr and co-authors report that plasma LECT2 concentration in normal individuals is 19.7 ± 3.4 ng/mL and generally rises in liver diseases, obesity, and insulin resistance [20]. In our population, the average concentration is more than twice that reported by Nasr and co-authors. The results do not indicate that elevated levels of LECT2 in our population are associated with liver disease, nor was there an association with BMI or insulin resistance, so we postulate that the panel of factors that contribute to elevated serum levels of this protein must be much larger than these three. It seems that the geriatric population generally has elevated LECT2 levels, although there is still no clear answer as to what the cause is.

The literature indicates that LECT2, as a chemotactic factor, can cause inflammatory cells to release inflammatory factors, thereby leading to vascular atherosclerosis. A study by Wei et al. shows a difference in the plasma levels of LECT2 in ACS (acute ischemic heart disease) and SAP (stable angina) patients and a higher level of LECT2 in those with myocardial infarction (MI) than those with unstable angina. The researchers proposed the thesis that LECT2 levels not only indicate the onset of atherosclerosis but may also correlate with atherosclerotic plaque stability. The study confirmed that patients with high LECT2 levels and myocardial infarction have a higher risk of cardiac death within 12 months than post-MI patients with low serum LECT2 levels [1]. Thus, it is possible to wonder about the diagnostic value of LECT2 as an auxiliary predictor in estimating the risk of death.

Since the findings suggest that the LECT2 protein participates in the development of inflammation, by the same reasoning, one would expect its steepness to increase with tissue superinfection and infection. Meanwhile, it turns out that plasma LECT2 concentrations in patients with sepsis were remarkably low and negatively correlated with the disease severity [20], which is rather the opposite of the expected effect. In atherosclerosis, inflammation plays a key role, leading to the destruction of the arterial endothelium and the initiation of atherosclerotic plaque formation and development; although it is sometimes initiated by a microbial agent, obviously, the pathomechanism of sepsis is quite different from atherosclerosis. Nevertheless, it is worth tracing the relationship between inflammatory proteins and LECT2 in the development of metabolic and senile diseases such as atherosclerosis and diabetes.

The rationale for LECT2 being involved in the development of diabetes was the discovery that deletion of the Lect2 gene in mice improves peripheral glucose entry into tissues [21]. In general, it was found that in both mice and humans, serum levels of LECT2 are increased in the case of insulin-resistant diabetes [21]. Lan et al. confirm that there is a correlation between LECT2 levels with the HOMA-IR insulin resistance index and glycated hemoglobin. A positive correlation is also found with skeletal muscle insulin resistance. Other studies have suggested that LECT2 also affects adipose tissue insulin resistance [22].

In the present project, we did not observe a correlation between LECT2 and any parameter directly related to diabetes (HbA1c, glycemia, presence of insulin resistance). SR-A, which correlates with LECT2 in our case, however, is indirectly related to diabetes because it is a receptor that may bind advanced glycation products. It is well known that overstimulation of AGEs is most often due to hyperglycemia, as elevated sugar levels create good conditions for glycooxidation, glycation, and oxidative stress. SR-A has a wide range of ligands and binds LDL in addition to AGEs, thus becoming a metabolite that stands at the intersection of lipid and sugar metabolism. Our uncovering of the SRA-LECT 2 relationship can thus be seen as the discovery of another common denominator for diabetes and atherosclerosis.

In the logical sequence, after discussing atherosclerosis and diabetes, is the metabolic syndrome, which links the two conditions. There are reports that LECT2 levels have a clear link to this syndrome. In a study of 200 people in Japan, serum LECT2 levels correlated positively with some clinical features of metabolic syndrome, namely body mass index, waist circumference, systolic blood pressure, serum selenoprotein P levels, and hemoglobin A1c blood levels [19]. In addition, Zhang’s studies have shown that serum levels of this protein correlate negatively with HDL-C while positively with triglyceride levels [23]. Our failure to obtain an association with any lipid profile parameter or any other parameter related to obesity, atherosclerosis, and metabolic syndrome may be due to either insufficient sample size or the fact that the geriatric group is governed by its own rules, and the aforementioned studies involved people in a wider age group, hence the differences. To verify what causes the differences in results between our project and others, this pilot study should be expanded to a much larger group.

The present study showed that the more LECT2, the lower the estimated glomerular filtration rate (EGFR) and probably more plasma creatinine. To the best of our knowledge, this is the first such report in the literature on a general population over 65 years of age. So far, the association of LECT2 and renal function has even been reported many times, but only in the context of one condition—leucocyte chemotactic factor 2 amyloidosis (ALECT2). It is estimated that ALECT2 is the third most common type of renal amyloidosis [24,25]. It is an acquired form of amyloidosis, although genetic factors also play a role-people with the I40V LECT2 gene polymorphism are particularly prone to ALECT2. Because of the prevalence of this form of polymorphism in the general population, it has been postulated that the onset of ALECT2 requires an additional genetically determined or hepatocyte damage-related increase in LECT2 expression [26]. Meanwhile, it is believed that the geriatric group in the context of kidney disease is mainly exposed to nephropathy as a complication of type 2 diabetes mellitus, and diagnosis is usually carried out in this direction. It is worth noting that the problem with the diagnosis of ALECT2 is that a kidney biopsy and histological examination must be performed to confirm it unequivocally. ALECT2 is thought to be a relatively benign type of renal amyloid associated with a slow decline in EGFR, but it is a relatively recently discovered disorder (2008), and many questions about the condition are still unanswered, including what exactly is the molecular basis of the process or what factors are underlying the apparent restriction of ALECT2 amyloidosis to some populations [24,25,26]. Our pilot study does not conclusively answer any of these questions and problems, but we believe that the demonstrated relationship between them is valuable information that completes the picture regarding renal function in the elderly. First of all, it should be verified whether in the geriatric population, there is indeed a widespread accumulation of LECT2 amyloid with the progression of aging or whether it is rather a marker of general deterioration of renal function.

## 5. Conclusions

In summary, we confirmed LECT2 expression in arteries and observed overexpression in atherosclerotic plaques. We confirmed significant statistical associations between LECT2 and SR-A levels and a negative correlation between LECT2 and EGFR.

We are aware that the main limitation of our study is the small sample size. Therefore, we consider this project as a prelude to a larger study on the pathomechanism of atherosclerosis, diabetes, and other degenerative processes associated with aging. As future research directions, we also plan to include in the analysis other promising non-routine markers of kidney function, such as cystatin C, β2-microglobulin (β2M), retinol-binding protein (RBP), neutrophil gelatinase-related lipocalin (NGAL) and others. It will also be important to verify the diagnostic value by expanding the project and analyzing LECT-2 to different age groups. We believe that in a large, adequately characterized study group, we will be able to trace cause-and-effect relationships and potentially draw conclusions regarding early diagnosis and prevention of conditions that contribute to premature patient death.

## Figures and Tables

**Table 1:** Correlation results between LECT2 and other blood parameters of the studied geriatric population. Parameters (r, *n*, *p*) in red for data where *p* < 0.05.

	r	*n*	*p*
	LECT2		
Glucose	−0.0578	32	0.753
HbA1c	−0.1992	25	0.340
Cholesterol	0.0175	31	0.925
HDL	0.0391	30	0.838
LDL	0.1019	30	0.592
Triglycerides	−0.0523	30	0.784
Creatinine	0.3508	31	0.053
Uric acid	0.0720	25	0.732
EGFR	−0.4122	31	0.021
AGEs	0.1050	33	0.561
GAL3	0.1496	33	0.406
LOX1	0.2293	33	0.199
sRAGE	0.1649	32	0.367
SR-B	0.2490	28	0.201
SR-A	0.6716	16	0.004

## Data Availability

The data presented in this study are available on request from the corresponding author. The data are not publicly available due to privacy restrictions.
